# PGC-1α Agonist Rescues Doxorubicin-Induced Cardiomyopathy by Mitigating the Oxidative Stress and Necroptosis

**DOI:** 10.3390/antiox12091720

**Published:** 2023-09-05

**Authors:** Shipra, Manoj Kumar Tembhre, Milind Padmakar Hote, Neetu Bhari, Ramakrishnan Lakshmy, S. Senthil Kumaran

**Affiliations:** 1 Department of Cardiac Biochemistry, AIIMS, New Delhi 110029, India; shipra@aiims.edu (S.);; 2 Department of Cardiothoracic & Vascular Surgery, AIIMS, New Delhi 110029, India; 3 Dermatology & Venereology, AIIMS, New Delhi 110029, India; 4 Department of N. M. R. (MRI Facility), AIIMS, New Delhi 110029, India

**Keywords:** cardiomyopathy, DCM, PGC-1α, oxidative stress, necroptosis, fibrosis

## Abstract

Cardiomyopathy (particularly dilated cardiomyopathy (DCM)) significantly contributes to development and progression of heart failure (HF), and inflammatory factors further deteriorate the symptoms. Morphological and functional defects of the heart in doxorubicin (DOX)-induced cardiomyopathy (cardiotoxicity) are similar to those of DCM. We used anagonist of PGC-1α (PPAR (peroxisome proliferator-activated receptor-gamma)-γ coactivator-1α) that is considered as the ‘master regulator’ of mitochondrial biogenesis with an aim to rescue the DOX-induced deleterious effects on the heart. Forty male C57BL/6J mice (8 weeks old) were divided in four groups, Control, DOX, ZLN005, and ZLN005 + DOX (*n* = 10 each group). The DOX-induced (10 mg/kg, single dose) cardiomyopathy mimics a DCM-like phenotype with marked morphologic alteration in cardiac tissue and functional derangements. Significant increased staining was observed for Masson Trichrome/Picrosirius red and α-Smooth Muscle Actinin (α-SMA) that indicated enhanced fibrosis in the DOX group compared to the control that was attenuated by (peroxisome proliferator-activated receptor-gamma (PPAR-γ) coactivator) (PGC)-1α (alpha) agonist (four doses of 2.5 mg/kg/dose; cumulative dose = 10 mg/kg). Similarly, elevated expression of necroptosis markers along with enhanced oxidative stress in the DOX group were alleviated by PGC-1α agonist. These data collectively suggested the potent therapeutic efficacy of PGC-1α agonist in mitigating the deleterious effects of DOX-induced cardiomyopathy, and it may be targeted in developing the future therapeutics for the management of DCM/HF.

## 1. Introduction

The majority of cardiomyopathy cases advance into heart failure, where terminal treatment is heart transplantation, which is challenging due to the limited availability of donors. Among cardiomyopathy cases, the largest prevalence is of the DCM phenotype, which accounts for more than 50% of heart transplantation cases [1] and is characterized by increased ventricular dimensions, thinning of chamber walls, reduction in ejection fraction, and overall compromised systolic function. DCM can be genetic, acquired, drug-induced, or idiopathic [2].

Owing to significant cardiotoxicity properties, the use of a chemotherapeutic drug like DOX is a major concern among cancer patients. The DOX-treated patients often presented with many life-threatening cardiovascular complications, prominent among which is cardiomyopathy (or DCM) [3]. Clinically, DOX-induced cardiomyopathy has similar morphological and functional derangements in the cardiac tissue compared to those of DCM which eventually culminate in HF. However, despite extensive research, the precise mechanism of DOX-induced cardiomyopathy (or DCM) is not yet completely understood. Cell death is a unifying event that occurs in the majority of heart injuries associated with disease conditions like ischemia-reperfusion, cardiomyopathy, myocardial infarction, and heart failure including DOX. The three well-known causative factors of cell death are (i) excessive oxidative stress due to massive production of reactive oxygen species (ROS); (ii) interaction of DOX with topoisomerase-α and -β by DNA (deoxyribonucleic acid) intercalation thereby causing double-strand breaks and hindering transcription machinery; (iii) mitochondrial damage [4,5,6,7,8].

In addition to apoptosis, there are various cell death mechanisms that have been explained as regulated cell death pathways, e.g., autophagy, necroptosis, pyroptosis, and ferroptosis [9,10]. In recent studies, the above-mentioned cell death pathways have been widely associated with DOX-induced cardiomyopathy [11] but which cell death pathway is predominantly triggered by DOX and the underlying mechanism is not clearly defined. Recently, necroptosis has gained wide attention and its role has been implicated in DOX-induced cardiomyopathy/cardiotoxicity [11,12,13]. Necroptosis has been identified as a cell death mechanism that occurred in a programmed way, and it is regulated by kinases, i.e., receptor-interacting protein kinases (RIPK1), RIPK3, and mixed-lineage kinase and domain-like pseudokinase (MLKL).

Furthermore, necroptotic cells release ‘alarmin’ molecules which act as danger signals for nearby cells [14,15]. Some of these molecules are ubiquitously present and have important roles in cellular functions, but the once outside cells, these act as danger signals for neighboring cells [14]. Alarmins are potent sources of inflammation and augment the recruitment of inflammatory immune cells and can also trigger cell death. Enhanced inflammatory milieu at the site of pathology augments oxidative stress leading to exacerbated tissue damage.

Therefore, strategies to annihilate the DOX-induced cardiomyopathy via regulating the necroptosis process, alarmin release, and reduction in oxidative stress are highly warranted, and the outcome of such studies may be applied in the management of different types of cardiomyopathies/cardiac injuries to prevent their progression towards HF. Targeting necroptosis resulted in protective effects on doxorubicin-induced cardiotoxicity models [9,10]. Considering the above facts, in this study, we targeted a nuclear-encoded transcriptional coactivator, i.e., PGC-1α which is considered as the ‘master regulator’ of mitochondrial biogenesis and its function. Furthermore, PGC-1α exerts a strong antioxidant effect [16], regulates cellular energy metabolism, cell growth [17], and its role has also been suggested in driving inflammation and immune regulation [18]. Activation of PGC-1α/SIRT (Sirtuin)1 pathway has been reported to be associated with elevation of autophagy/mitophagy leading to suppression of oxidative stress-mediated ROS production [19]. However, the role of PGC-1α in regulating necroptosis, alarmin production, and oxidative stress in a doxorubicin-induced cardiomyopathy model has not been described. Therefore, the present study employed PGC-1α agonist (i.e., ZLN005) with an aim to rescue the DOX-induced deleterious effects on the heart via alleviating the necroptosis and oxidative stress, thereby restoring the cardiac function by preventing extensive cardiac tissue remodeling.

## 2. Materials and Methods

### 2.1. Mice and Experimental Procedures

All animal experiments were approved by Institute Animal Ethics Committee, All India Institute of Medical Sciences (AIIMS), New Delhi. C57BL/6J male mice of 24–27 g (8 weeks old) were purchased from Central Animal Facility, AIIMS, Delhi. Mice were fed ad libitum and were housed under standard 12 h light–dark cycles with controlled temperature and humidity in ventilated rooms. Daily performance of mice, their weight, and behavior were recorded. At the end of the treatment regime, mice were euthanized humanely with overdose of isoflurane. The hearts were perfused, isolated, and used for subsequent experiments. Forty mice were randomly divided into four groups, i.e., control, DOX, ZLN005, and ZLN005 + DOX (n = 10, each group). The doxorubicin model was established as previously described [20]. Briefly, doxorubicin (single dose, 10 mg/kg, i.p., Cayman Chemicals, Ann Arbor, MI, USA) was administered in DOX group and survival schedule of 7 days was followed. Control mice were administered with vehicle (1X PBS, i.p.). The ZLN005 + DOX group received DOX and ZLN005 whereas ZLN005 group received ZLN005 only. Repeated dosing model was adapted for ZLN005-based intervention [21]. The ZLN005 (four doses of 2.5 mg/kg/dose, cumulative dose, 10 mg/kg, Cayman Chemicals, Ann Arbor, MI, USA) was administered i.v., 2 h after doxorubicin [22] through retro-orbital route every alternate day as demonstrated schematically in result section.

### 2.2. Gene Expression by Quantitative PCR (qPCR)

Heart tissues were homogenized using mortar and pestle in liquid nitrogen and transferred to TriExtract (G-Biosciences, St. Louis, MO, USA). Total RNA was isolated and quantified followed by DNase treatment (DNase I, RNase-free, ThermoFisher Scientific, Vilnius, VA, Lithuania). Complementary DNA (cDNA) was synthesized using cDNA synthesis kit (iScript cDNA synthesis kit, BioRad, Hercules, CA, USA) following manufacturer’s instructions. The PCR was performed using iTaq Universal SYBR Green Supermix (BioRad, Hercules, CA, USA) in CFX96 Real-time PCR system, (BioRad, Hercules, CA, USA) in accordance with MIQE guidelines. PCR cycles were set for 10 min denaturation, followed by 40 cycles of denaturation at 95 °C for 15 s, followed by primer annealing and extension at optimized temperature. Samples were run in triplicates and GAPDH (Glyceraldehyde 3-phosphate dehydrogenase) was used as housekeeping gene for normalization of data. The primers used in our study include *CTSB* (Cathepsin-B), *HMGB1* (High Mobility Group Box-1), *RIPK1*, *RIPK3*, *MLKL*, *S100b* (S100 calcium binding protein-B) and *PGC-1α*. The list of primer sequences is mentioned in Table 1. The gene expression data were represented as 2^−∆Ct^ for all the groups.

### 2.3. Immunofluorescence

Freshly isolated mouse hearts were dissected on ice and immediately fixed in 4% paraformaldehyde. Tissue was paraffin-embedded and sectioned at 3–5 microns. The sections were deparaffinized followed by antigen retrieval and blocking. Primary antibody incubation with RIPK1 (H-207) (1:500, Santa Cruz Biotechnology, Santa Cruz, CA, USA), RIPK3 (H-43) (1:500, Santa Cruz Biotechnology, Santa Cruz, CA, USA), MLKL (1:500, Thermo Fisher Scientific, Waltham, MA, USA), alpha-SMA (1: 500, Abcam, Cambridge, UK), PGC-1α (1:200, ABclonal, Woburn, MA, USA) at 4 °C was incubated overnight, followed by washing and incubation with FITC-conjugated goat anti-rabbit (1:500, Abcam) secondary antibody for one hour at room temperature, followed by nuclei counterstain with 4′,6-diamidino-2-pheylindole (DAPI) (Sigma Aldrich, St. Louis, MO, USA). Tissue sections were washed and mounted using Vectashield antifade mounting medium (Vector Laboratories, Newark, CA, USA). Images were captured using Nikon Eclipse Ni upright fluorescent microscope (Nikon, Tokyo, Japan) using NIS-Elements Br software, Ver. 4.30 (Nikon, Tokyo, Japan). Mean fluorescence intensity of signal and background was calculated using Fiji software, ImageJ 2.3.0/1.53r, Java 1.8.0_322 (64-bit) (NIH, Bethesda, MD, USA). The data from 4 different sections were used to capture five different fields of views each, which were used for further statistical analysis.

### 2.4. Histological Analysis

For Trichrome staining, sections were deparaffined in decreasing gradient of ethanol, fixed in Bouin’s solution (Sisco Research Laboratories Pvt. Ltd., Mumbai, Maharashtra, India) overnight. Sections were washed in running tap water followed by staining of nucleus using modified Weigert’s Iron hematoxylin stain (Sisco Research Laboratories Pvt. Ltd., Mumbai, Maharashtra, India) which was washed, and staining was proceeded for trichrome stain to discriminate between normal tissue from fibrotic (collagen deposition appeared as blue stain), followed by dehydration and mounting. Similarly, for Picrosirius Red staining, the deparaffinized tissue sections were stained using modified Weigert’s hematoxylin (Sisco Research Laboratories Pvt. Ltd., Mumbai, Maharashtra, India)which was washed to stain for Sirius Red (Sisco Research Laboratories Pvt. Ltd., Mumbai, Maharashtra, India) for one hour, which was proceeded by dehydration and (collagen deposition appeared as red stain)mounting of tissue using DPX Mountant (Sigma, Burlington, MA, USA).

### 2.5. Protein Quantification by Enzyme Linked Immune Sorbent Assay (ELISA)

Protein quantification was performed using ELISA. Mouse 8-hydroxy-desoxygaunosine (8-OHdG)(MyBioSource, San Diego, CA, USA), Malondialdehyde (MDA) (Elabscience, Houston, TX, USA), Catalase (MyBioSource, San Diego, CA, USA), Superoxide Dismutase (MyBioSource, San Diego, CA, USA), Glutathione (GSH, MyBioSource, San Diego, CA, USA), Glutathione Reductase (GSSG, (MyBioSource, San Diego, CA, USA), Collagen-I (MyBioSource, San Diego, CA, USA) Collagen III (MyBioSource, San Diego, CA, USA), Fibronectin (MyBioSource, San Diego, CA, USA), and Laminin (MyBioSource, San Diego, CA, USA) kits were used and all the immunoassay steps were performed as per manufacturers’ instructions. Optical density was measured using Epoch microplate reader (BioTek, Winooski, VT, USA; Agilent, Santa Clara, CA, USA) at 450 nm. The concentrations were expressed as picogram (pg) or nanogram (ng) per milliliter (mL).

### 2.6. Statistical Analysis

All data were analyzed using GraphPad Prism 8 (GraphPad Software, Inc., San Diego, CA, USA). Data were analyzed for normality using D’Agostino and Pearson test, Shapiro–Wilk test, Kolmogorov–Smirnov test, and visually validated by plotting Q-Q plot. Mann–Whitney U test was conducted for non-parametric tests (qPCR data). One-way ANOVA (ELISA data) and unpaired t test (Immunofluorescence data quantification) were performed for normally distributed data. Significance test was performed using *p*-value of <0.05, error bars indicated mean ± SD (standard deviation). All experiments were performed in triplicates and the average was used for subsequent analysis.

## 3. Results

**Figure 1:**
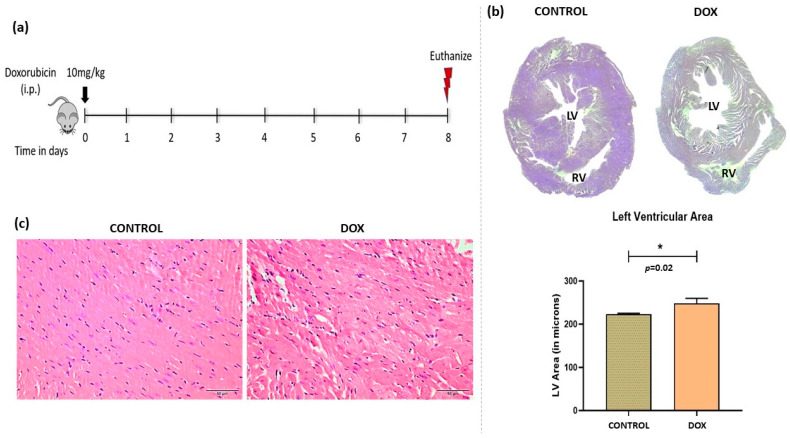
Doxorubicin-induced model of cardiomyopathy. (**a**) Schematic diagram of dose schedule for doxorubicin-induced cardiomyopathy model. (**b**) Micrograph of cardiac tissue (**upper right panel**) and quantification of left ventricular area (**lower right panel**). (**c**) Hematoxylin and eosin staining of control and doxorubicin-treated heart. LV = Left Ventricle, RV = Right Ventricle. Scale bar = 50 µm, (*) = *p* < 0.05.

Doxorubicin is a widely used chemotherapeutic drug. It demonstrates dose-dependent toxicity. We have used an acute cardiotoxicity model with short-term survival (Figure 1a). Overall, the heart showed enhanced dilatation of chambers and higher filling capacity as quantified by histology images (Figure 1b). Alterations in cardiac tissue morphology showed enhanced vacuolations, distorted myofibril structure, and increased leukocyte infiltration (Figure 1c).

**Figure 2:**
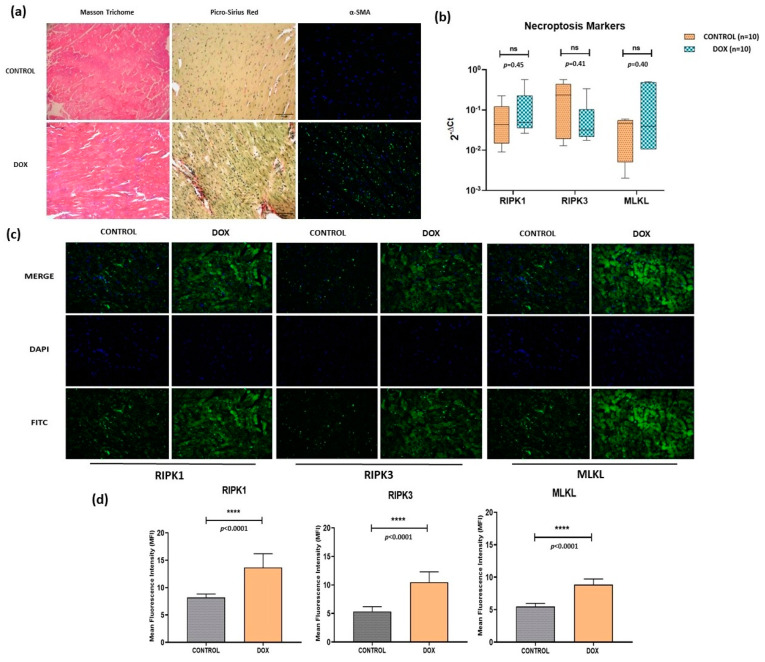
(**a**) Analysis of cardiac tissue fibrosis via Masson Trichrome Staining (muscle tissue = pink to red, collagen deposition = blue and nucleus = black), Picro-Sirius Red staining (muscle tissue, cell cytoplasm = yellow, collagen deposits = red and nucleus = black), and α-SMA (blue = nucleus and green = α-SMA). Scale = 50 μm. (**b**) qPCR-based transcripts expression for necroptosis markers (*RIPK1*, *RIPK3*, *MLKL*). (**c**) Immunofluorescence of necroptosis markers (RIPK1, RIPK3 and MLKL) in control and doxorubicin-treated groups. Magnification = 40×. (**d**) Quantification of immunofluorescence data. ns = non-significant, (****) = *p* < 0.0001.

We analyzed fibrosis in cardiac tissue by Masson Trichrome, Sirius Red, and α-SMA (alpha-smooth muscle actin) staining, and significantly increased staining revealed enhanced fibrosis by all the three methods in the DOX group compared to controls (Figure 2a). Furthermore, necroptosis cell death pathway was analyzed by immunofluorescence-based protein localization (and quantification) for key molecules like RIPK1, RIPK3, and MLKL in cardiac tissue. Significantly increased staining (*p* < 0.0001) was observed for all three markers in the DOX group compared to controls which was indicative of enhanced necroptosis (Figure 2c,d). However, corresponding mRNA levels showed differential expression patterns that were not statistically significant (Figure 2c).

**Figure 3:**
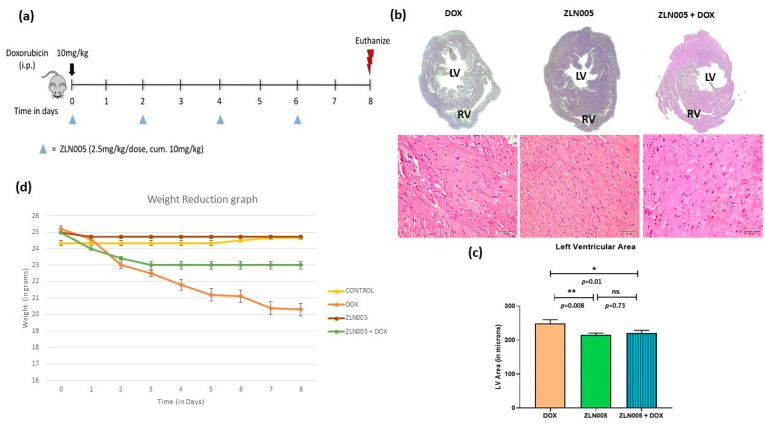
PGC-1α agonist (ZLN005)-based intervention to rescue doxorubicin-induced model of cardiomyopathy. (**a**) Schematic diagram of dose schedule for ZLN005-based intervention in doxorubicin-induced cardiomyopathy model. (**b**) Micrograph of cardiac tissue (**top**) and hematoxylin and eosin staining (**bottom**) of doxorubicin-treated heart and intervention group. Scale bar = 50 µm. (**c**) Quantification of left ventricular area. (**d**) Comparison of body weights of mice with time (in days) in all the study groups. LV = Left Ventricle, RV = Right Ventricle. ns = non-significant, (*) = *p* < 0.05, (**) = *p* < 0.01.

**Figure 4:**
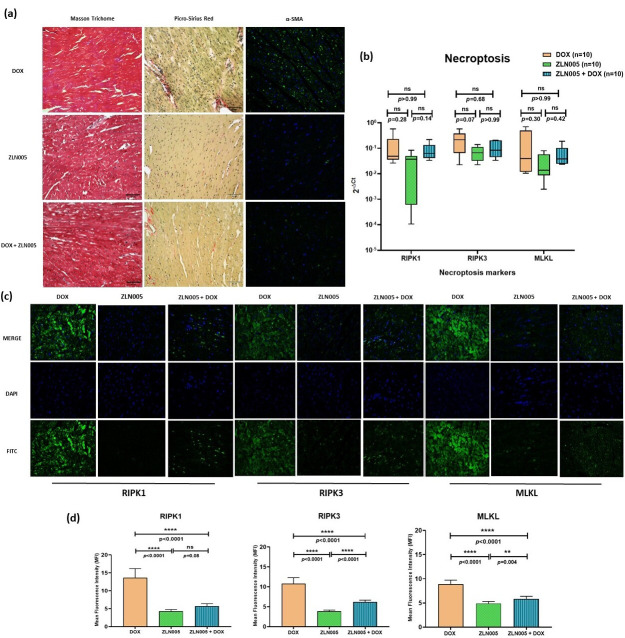
(**a**) Analysis of fibrosis via Masson Trichrome Staining (muscle tissue = pink to red, collagen = blue and nucleus = black), Picro-Sirius Red staining (muscle tissue, cell cytoplasm = yellow, collagen = red and nucleus = black), and α-SMA (blue = nucleus and green = staining of α-SMA) in DOX, ZLN005, and ZLN005 + DOX groups. Scale bar = 50 μm. (**b**) qPCR-based transcripts expression for necroptosis markers (RIPK1, RIPK3, MLKL). (**c**) Immunofluorescence of necroptosis markers (RIPK1, RIPK3, and MLKL) in study groups. Magnification = 40×. (**d**) Quantification of immunofluorescence data. ns = non-significant, (**) = *p* < 0.01, (****) = *p* < 0.0001.

**Figure 5:**
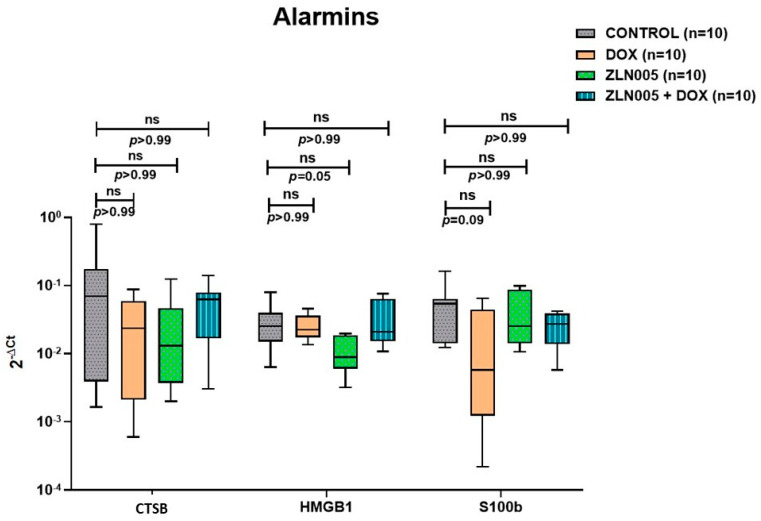
Transcript expression levels (qPCR) of alarmin molecules in control, DOX, ZLN005 and ZLN005 + Dox hearts. *CTSB* = Cathepsin-B, *HMGB1* = High Mobility Group Box-1, *S100b* = S100 calcium binding protein B, ns = non-significant (significant *p* value is set as *p* < 0.05).

We identified PGC-1α agonist, i.e., ZLN005, which significantly increased the expression of PGC-1α protein and transcript levels in the heart tissue of ZLN005 and ZLN005 + DOX groups compared to DOX group (Figure S1, supplementary data). In order to rescue the DOX-induced cardiomyopathy phenotype, we treated mice with ZLN005 as mentioned in the above section (Figure 3a). We observed remarkable recovery in myocardial mass, reduced dilatation of heart and vacuolations with overall improvement in structural integrity in ZLN005 + DOX group compared to the DOX group (Figure 3b,c). Weight reduction, a critical parameter to determine the overall health and metabolism of mice, also revealed significant improvement in ZLN005 + DOX group when compared to the doxorubicin-treated group (Figure 3d). Furthermore, a marked decrease in Masson’s Trichrome, Sirius Red, and α-SMA stainings (Figure 4a) were observed in ZLN005 + DOX treated group compared to the DOX group. Significant reduction (*p* < 0.0001) in immunofluorescence-based protein localization of necroptosis markers was observed in the ZLN005 + DOX-treated group compared to the DOX group, indicating enhanced survival of cardiomyocytes attributed by PGC-1α agonist (Figure 4c,d). However, no significant difference was observed in the corresponding transcript levels when all the three groups were compared (Figure 4b). Next, the transcript expression levels of alarmin molecules, i.e., *HMGB1*, *S100b*, and Cathepsin-B (*CTSB*) were determined, but statistically significant difference was not observed for all three studied genes in all the four groups (Figure 5).

**Figure 6:**
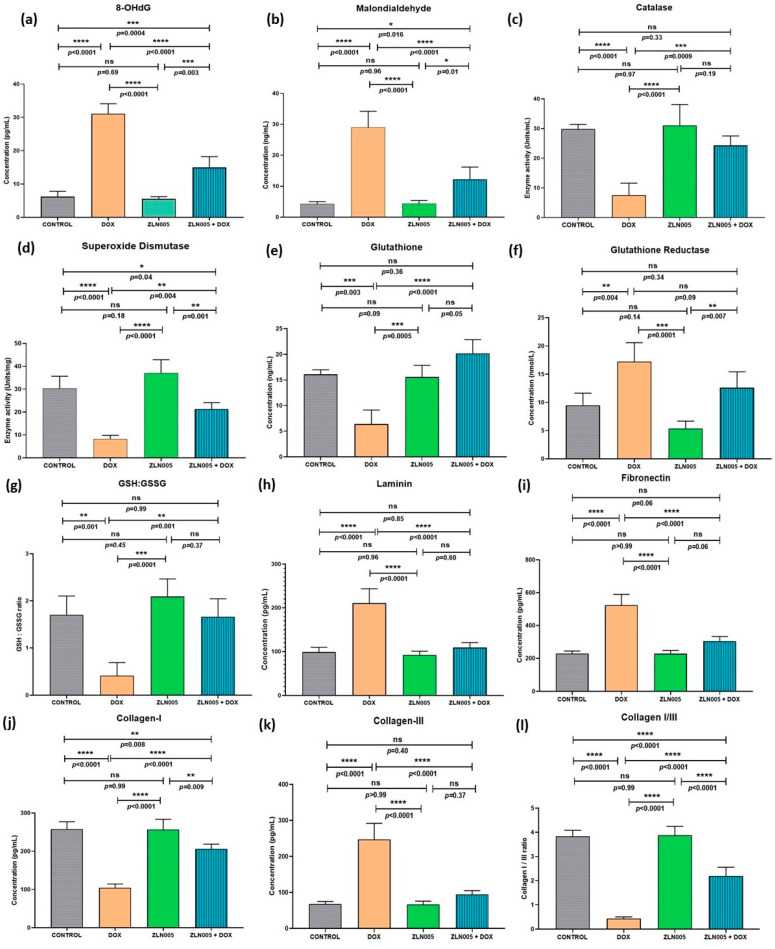
(**a**–**g**) Status of oxidative stress markers in cell lysates of cardiac tissue of control, DOX, ZLN005, and ZLN005 + DOX groups. (**h**–**l**) Tissue remodeling parameters for cardiac cell lysates of all the study groups. 8-OHdG = (8-oxo-7,8-dihydro-2′-deoxyguanosine), GSH: GSSG = ratio of glutathione to glutathione reductase. (*) = *p* < 0.05, (**) = *p* < 0.01, (***) = *p* < 0.001, (****) = *p* < 0.0001.

Since DOX triggered a potent oxidative stress response, we further investigated the status of hallmark markers associated with oxidative stress response to understand the ZLN005 effect. We analyzed the levels of 8-OHdG (a marker of DNA damage) (Figure 6a,b) and MDA (a lipid peroxidation marker) that were found to be significantly increased in the DOX group compared to controls (*p* < 0.0001), and corresponding levels were significantly reduced in ZLN005 + DOX (*p* = 0.0004 for 8-OHdG and *p* = 0.016 for MDA) but remained higher compared to the control group. Conversely, the ROS (reactive oxygen species)-scavenging enzyme, i.e., SOD and catalase activities, were significantly decreased in the DOX group compared to the control (*p* < 0.0001), and activities of the same were increased after ZLN005 treatment (DOX + ZLN005 group) but they were comparable to the control group (Figure 6c,d). Similarly, glutathione (GSH) levels and Glutathione Reductase (GSSG) were respectively decreased (*p* = 0.003) and increased (*p* = 0.004) in the DOX group compared to the control, while contrasting trends were observed in the DOX + ZLN005 group (Figure 6e,f) compared to the DOX group. Likewise, the GSH:GSSG ratio was decreased in the DOX group compared to the control (*p* = 0.001) and it was restored to normal in the DOX + ZLN005 group (*p* = 0.001) (Figure 6g).

Based on high α-SMA and fibrosis staining that indicated significant tissue remodeling, we further investigated the effect on cardiac tissue remodeling by quantifying the levels of key extracellular matrix proteins involved in structural homeostasis, i.e., laminin, fibronectin, collagen I, and collagen III. We observed significant upregulation of laminin and fibronectin in the DOX group compared to the control group (*p* < 0.0001), but their levels remained comparable to control in the ZLN005 + DOX group (Figure 6h,i). Next, as expected, the collagen-I and collagen III were significantly decreased and increased, respectively, in the DOX group (*p* < 0.0001) compared to controls, but these alterations were preserved by ZLN005 treatment as evident by ratio of collagen I to collagen III (Figure 6l). In all the above-studied parameters, the control groups were found comparable to the ZLN005 group.

## 4. Discussion

Heart failure (HF) remains a global health issue that requires development of newer treatment strategies to combat the alarmingly increasing disease burden. As mentioned above, a significant proportion of cancer survivors who received chemotherapy (e.g., DOX) were also presented with various types of cardiomyopathies [4,5,6] and at present, dexrazoxane is the only clinically approved agent used to minimize the DOX-induced cardiotoxicity [12].

Since DOX causes mitochondrial dysfunction and mitochondrial homeostasis is critical to meet the high energy demand of a dynamic organ like the heart [23,24], it is imperative to target a molecule that will compensate the DOX-mediated toxic effect on mitochondrial dynamics along with reducing the oxidative stress and cell death.

As described earlier, PGC-1α is a master regulator of mitochondrial biogenesis and it is also essential for the cardiomyocytes during developmental stages. It has been reported as a crucial factor for differentiation/maturation of human embryonic stem cells (hESC) into cardiomyocyte phenotype [25] and it is also known to regulate the respiration process in hiPSC (human-induced pluripotent stem cells)-generated cardiomyocytes [26]. The role of PGC-1α in mitigating cardiac injury is, however, not well defined particularly in context with necroptosis, oxidative stress, and cardiac tissue remodeling. Decreased PGC-1α expression is reported in doxorubicin-induced model of cardiotoxicity [8] and in vitro models of cardiac hypertrophy [27]. Furthermore, enhancing PGC-1α in a renal I/R injury model was able to reverse fibrosis [28] and rescue nephrotoxicity. Similar observations were observed in a neurotoxicity I/R model [22]. Liang et al. reported that the oxidative damage and mitochondrial dysfunction induced by H_2_O_2_ exposure in IPEC (Intestinal porcine epithelial cells)-1 intestinal cells has been ameliorated by activation of the PGC-1α/SIRT1 pathway via upregulating autophagy/mitophagy [19], indicating the critical role of PGC-1α in regulating oxidative stress and mitochondrial function. Therefore, we employed PGC-1α agonist with an aim to rescue the DOX-induced cardiomyopathy-like phenotype. Our findings revealed an increased cardiac tissue mass and improved structural changes with reduced vacuolations and fibrosis pointing towards overall improvement in DOX-induced injury, and the data were found in agreement with the above-mentioned studies in different disease models. PGC-1α possesses potent anti-oxidant properties, and its role has been demonstrated in regulating the cellular growth and mitochondrial antioxidant defense system in distinct cell types under various stressors’ influence/disease conditions [16,19,29,30]. In the present study, ZLN005 treatment mitigated the harmful effects of DOX-induced oxidative stress (kept the status quo with the control group) with corresponding improvement in cardiac fibrosis and preserved structural integrity.

Enhanced activities of free radical scavenging enzymes, i.e., catalase, SOD and GSH and decreased concentration of 8-OHdG (DNA damage marker), MDA (lipid peroxidation marker), and GSSH were observed in the ZLN005 intervention (ZLN005 + DOX) group compared to the DOX group. Further, the levels of catalase, glutathione, glutathione reductase, and GSH:GSSG ratio of the ZLN005 + DOX group was found comparable to the control group.

Moreover, 8-OHdG increases with oxidative stress and ROS accumulation, thereby contributing to DNA damage and is related to cardiovascular disease onset and progression [31,32]. The status of key antioxidant enzymes including catalase, glutathione, and superoxide dismutase were enhanced in the ZLN005 + DOX group. Catalase overexpression in the heart prevents progression into heart failure and influences myocardial remodeling by reducing fibrosis [33]. The SOD serves as the first line of defense against ROS and SOD levels are related to adverse LV geometry and progression towards HF [34] and similar findings were observed in the DOX model of the present study. Increased SOD levels are associated with cardioprotective effect [35], and similar outcomes were observed in the ZLN005 + DOX group.

Alarmins are a set of ubiquitously present molecules mediating diverse physiological roles. Alarmins may play a dual role that can be protective or detrimental for cells in a spatio-temporal context. Cell survival and proliferation is regulated by S100b; it has been shown to promote neuronal cells’ survival in picomolar to nanomolar quantities and cell death in micromolar concentrations [36]. Further, stimulation with S100b inhibits myogenic differentiation [37] and post-DOX treatment cardiomyocytes have a tendency to differentiate into fibroblast-like phenotype with a corresponding decrease in S100b levels. Intracellular S100b reportedly inhibited apoptosis in myoblasts while playing a role in their differentiation [38]. In our present study, we observed similar trends, where *S100b* transcript levels were decreased in the DOX group and increased after ZLN005 (ZLN005 + DOX group) treatment but the comparison was not statistically significant. HMGB1 is another alarmin investigated and is responsible for mediating cardiac injury [39,40]. In contrast to our study, i.e., DOX (10mg/kg, i.p.) with 1 week survival, Yao et al. administered DOX (20 mg/kg, i.p.) with survival of 5 days [40] where they found increased HMGB1 protein expression in cardiac tissue along with increased concentration of circulating HMGB1 in serum of the DOX group compared to the control group. Another study demonstrated a differential binding pattern of HMGB1 to DNA with a dose-dependent response of DOX, i.e., increased HMGB1-DNA binding at low DOX concentration and vice versa [41]. Cathepsin–B is a lysosomal protease that is associated with aggravation of symptoms in the DOX injury model [42]. Liu et al. reported increased Cathepsin-B protein expression in cardiomyocyte cell line H9C2, post DOX (0.5 μM, 24 h) treatment [42]. Cathepsin-B is also known to mediate cardiac remodeling events [43] and play a role in the progression of cardiomyopathy phenotype through cell death pathways [44]. However, in our study, we observed no significant variation in transcript expression levels of *HMGB1* and *CTSB* genes in cardiac tissue and corresponding protein levels will be required to address these discrepancies.

During extracellular matrix remodeling, components like fibronectin and laminin play an important role in deposition of collagen fibers within the myocardium. This leads to extracellular matrix protein accumulation leading to stiffening of the myocardium. Inhibition of fibronectin improves cardiac dysfunction and halts progression towards heart failure [45]. In our present study, we found significantly increased concentration of laminin and fibronectin proteins in heart tissue lysates in the DOX group and it was restored to normal levels after ZLN005 treatment thereby circumventing the harmful DOX-induced tissue remodeling process. Furthermore, Col I/III ratio is an important factor involved in tissue remodeling associated with myocardial infarction and progression toward HF [46,47]. In the present study, we found a decreased Col I/III ratio in the DOX group compared to the control. The ZLN005 (ZLN005 + DOX group) treatment improved the Col I/III ratio via skewing it towards the control. All these data collectively demonstrated the potential tissue remodeling effect of PGC-1α that has not been reported previously.

The present study does not report data related to cardiac function. The echocardiography-based cardiac function data (e.g., ejection fraction (EF), LVEDD (left ventricular end-diastolic dimension), LVESD (left ventricle end-systolic dimension), FS (fractional shortening,) etc.) would be of great interest to understand the effect of PGC-1α agonist on the functional cardiac parameters, and it is one of the limitations of the present study.

## 5. Conclusions

**Figure 7:**
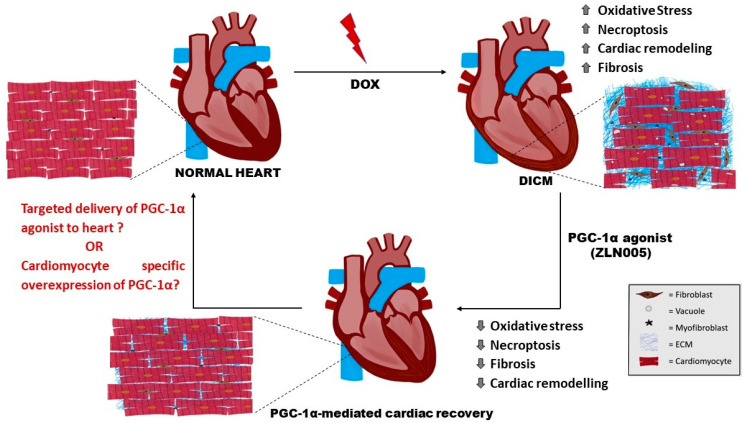
Schematic representation of DOX-induced cardiotoxic effects (enhanced oxidative stress, necroptosis, fibrosis, and cardiac remodeling) which were mitigated by PGC-1α agonist, i.e., ZLN005. (? = require further investigation). DOX= doxorubicin, DICM = DOX-induced cardiomyopathy, ECM = Extracellular Matrix.

In summary, this study described the role of PGC-1α agonist (ZLN005) in mitigating cardiomyopathy phenotype by strengthening the redox balance via mitigating the DOX-mediated oxidative stress, preventing the harmful tissue remodeling effects and necroptosis. However, a comprehensive study is required to understand the holistic effect of PGC-1α agonist and PGC-1α-mediated regulatory effects on the heart. As described in Figure 7, a targeted delivery of PGC-1α agonist in the heart or cardiomyocyte-specific overexpression of PGC-1α may enhance the therapeutic outcome in the present experimental set-up. The outcome may be translated to the clinical set-up for the management of various cardiac injury-based disease conditions and/or to prevent their progression towards HF.

## Figures and Tables

**Table 1:** List of genes and primers.

S.No.	Gene Name	Accession ID	Forward Primer (5′→3′) Reverse Primer (5′→3′)
1.	*CTSB*	NM_007798.3	GGCTCTTGTTGGGCATTTGG
			CAGCTTCACAGCTCTTGTTGC
2.	*HMGB1*	NM_001313894.1	TCCCTCATCCTTGTTTACTCG
			GCAGTTTCCTATCGCTTTGG
3.	*RIPK1*	NM_001359997.1	AGGTGTCCTTGTGTTACC
			CCTCCACGATTATCCTTCC
4.	*RIPK3*	NM_019955.2	AAGACAGTCCTTGCCACTTCC
			TGGGTCAAGAGTCAGTTTGGG
5.	*MLKL*	NM_001310613.1	ATGCCAGCGTCTAGGAAACC
			TCGGGCAGGTTCTTCTTTCC
6.	*S100B*	NM_009115.3	ACAACGAGCTCTCTCACTTCC
			CATCTTCGTCCAGCGTCTCC
7.	*PGC-1α*	NM_008904.3	GCACACACCGCAATTCTCC
			AGGCTTCATAGCTGTCGTACC
8.	*GAPDH*	NM_001411843.1	AACTTTGGCATTGTGGAAGGG
			CATCACGCCACAGCTTTCC

## Data Availability

Not applicable.

## Supplementary Materials

The following supporting information can be downloaded at: https://www.mdpi.com/article/10.3390/antiox12091720/s1, Figure S1: (a) Immunofluorescence of PGC-1α expression in control, DOX, ZLN005 and ZLN005+DOX. (b) Quantification of Immunofluorescence data. (c) Transcript expression of PGC-1α in Control, DOX, ZLN005 and ZLN005+DOX groups. (*) = *p* < 0.05, (**) = *p* < 0.01, (****) = *p* < 0.0001, ns = non-significant (significant *p* value is set as *p* < 0.05).
