# Supplementary material for: PGC-1α Agonist Rescues Doxorubicin-Induced Cardiomyopathy by Mitigating the Oxidative Stress and Necroptosis
Source: Antioxidants (Basel). 2023 Sep 5;12(9):1720. doi: 10.3390/antiox12091720 (PMC10525725; doi:10.3390/antiox12091720)
Supplement: Supplementary file 1 [file antioxidants-12-01720-s001.zip › antioxidants-2508416-supplementary.pdf]

Supplementary Materials: PGC1α Agonist Rescues Doxorubicin Induced Cardiomyopathy by Mitigating the Oxidative Stress and Necroptosis

Shipra Shipra [1], Manoj Kumar Tembhre [1,*], Milind Padmakar Hote [3], Neetu Bhari, Ramakrishna Lakshmy [1] and S. Senthil Kumaran [4]

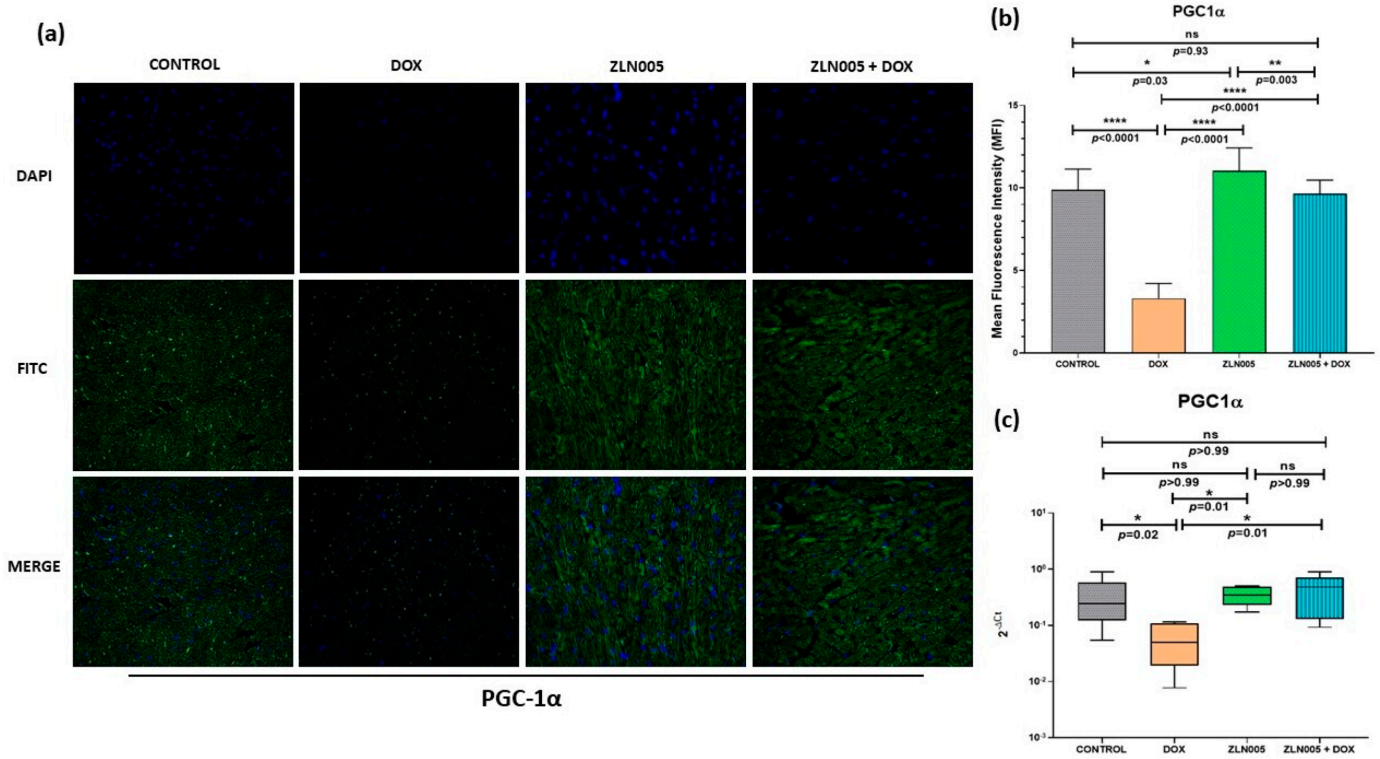

Figure S1. (**a**) Immunofluorescence of PGC1α expression in control, DOX, ZLN005 and ZLN005+DOX. (**b**) Quantification of Immunofluorescence data. (**c**) Transcript expression of PGC1α in Control, DOX, ZLN005 and ZLN005+DOX groups. ns= non-significant (significant p value is set as $p < 0.05$), (*)= $p<0.05$, (**)=$p<0.01$, (***)=$p<0.001$, (****)=$p<0.0001$.
